# Supplementary material for: Therapeutic Effect of C-Vx Substance in K18-hACE2 Transgenic Mice Infected with SARS-CoV-2
Source: Int J Mol Sci. 2023 Jul 26;24(15):11957. doi: 10.3390/ijms241511957 (PMC10418837; doi:10.3390/ijms241511957)
Supplement: Supplementary file 1 [file ijms-24-11957-s001.zip › ijms-2484246-supplementary-updated.pdf]

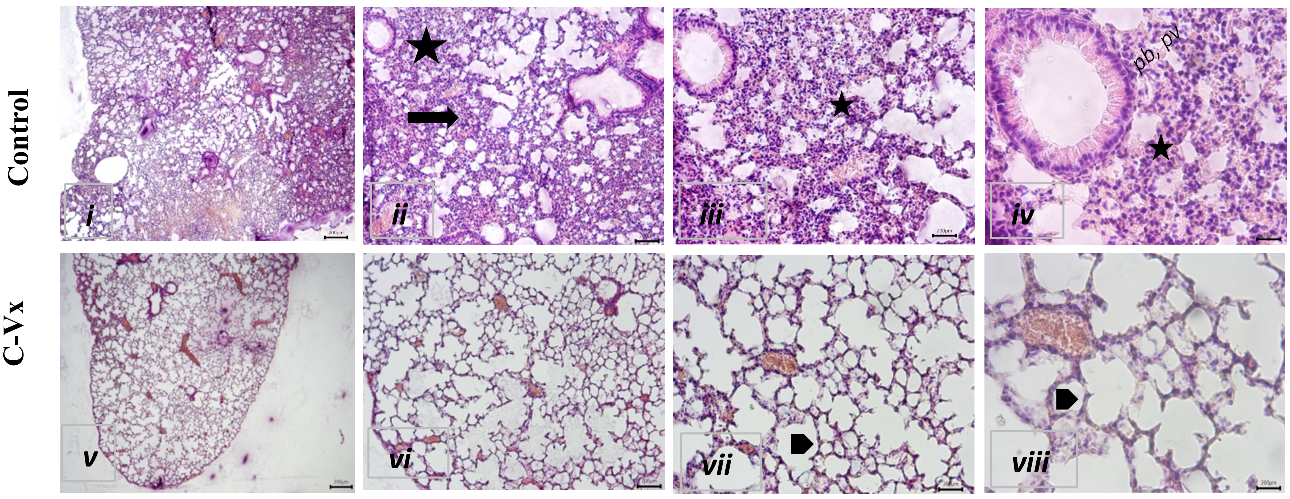

Supplement Figure 1: The IHC graph of all mice and several representative IHC images of lung tissue from the control (*i*) and C-Vx (*ii)* groups. A) The tissue architecture of control group shows interstitial inflammatory cell infiltration ***(asterisk, ii, iii and iv)*,** alveolar septal thickening *(arrow, ii)* and more parenchymal infiltration at **peribronchiolar *(pb, iv)* and perivascular *(pv, iv)* regions**. Vaccinated C-Vx group shows decreased amount of interstitial inflammatory cell infiltration and normal alveolar morphology *(arrowhead, vii, viii)*. Image magnification of *i* and v are 4x, *ii* and *vi* are 10x, *iii* and *vii* are 20x, *iv* and *viii* are 40x. **Scale bar: 200 µm.**

Supplementary Table 1: Expression of SARS-CoV-2 virus nucleocapsid gene by RT-PCR and weight changes of animals (N/A: Not applicable, EX: exitus).

Dose Studies	Groups	Animal No	Weight change (%)	Pulmonar Tissue SARS-CoV-2 RT-PCR Ct	
				N1	N2
Study 1	Control	1	-15.58	N/A	22
		2	-14.55	EX	EX
		3	6.38	32	26
		4	-28.95	EX	EX
	Group 1 100 µL/kg	1	-18.16	EX	EX
		2	-14.63	EX	EX
		3	2.78	N/A	N/A
		4	-22.61	EX	EX
	Group 2 130 µL/kg	1	-24.47	EX	EX
		2	-29.00	EX	EX
		3	-0.84	N/A	N/A
		4	0.00	27	26
	Group 3 180 µL/kg	1	0.53	35	35
		2	1.25	19	19
		3	-3.08	19	19
		4	-4.78	19	20
Study 2	Control	1	-20.33	18	16
		2	-16.55	16	15
		3	-14.86	17	16
		4	-21.92	18	17
		5	-14.51	17	16
		6	-2.84	N/A	N/A
	Group 1 180 µL/kg	1	-11.09	23	22
		2	-1.69	N/A	40
		3	-9.41	21	20
		4	-16.00	24	24
		5	-2.44	N/A	N/A
		6	-19.12	24	23
	Group 2 220 µL/kg	1	-3.40	N/A	N/A
		2	-34.97	19	21
		3	-22.44	19	19
		4	-4.07	N/A	40
		5	1.09	N/A	N/A
		6	-1.23	N/A	N/A
	Group 3 250 µL/kg	1	-1.07	22	21
		2	-1.15	22	21
		3	-3.23	22	22
		4	-27.94	18	17
		5	-4.91	19	20
		6	-0.71	19	18
	Control	1	-32.66	21	23
		2	-24.66	23	24

Study 3		3	-38.40	26	25
		4	-36.92	26	25
		5	-26.30	EX	EX
		6	-14.26	24	23
		7	-9.41	18	17
		8	-7.43	21	21
		9	-7.96	23	24
		10	-22.56	37	36
	Group 1 250 μL/kg	1	3	N/A	N/A
		2	1.64	N/A	N/A
		3	5.22	N/A	N/A
		4	0.99	N/A	N/A
		5	-34.25	16	18
		6	-7.22	N/A	N/A
		7	1.88	N/A	N/A
		8	-16.03	N/A	N/A
		9	11.45	N/A	N/A
		10	1.33	N/A	N/A
